# Issues in the Development of a Research and Education Framework for One Health

**DOI:** 10.3201/eid1903.121103

**Published:** 2013-03

**Authors:** Lisa M. Gargano, Patrick F. Gallagher, Meredith Barrett, Kelly Howell, Cameron Wolfe, Christopher Woods, James M. Hughes

**Affiliations:** Author affiliations: Emory University School of Medicine, Atlanta, Georgia, USA (L.M. Gargano, K. Howell, J.M. Hughes);; Emory University Rollins School of Public Health, Atlanta (P.F. Gallagher);; University of California, San Francisco, California, USA (M. Barrett); University of California, Berkeley, California, USA (M. Barrett); Duke University School of Medicine, Durham, North Carolina, USA (C. Wolfe, C. Woods)

**Keywords:** One Health, zoonoses, environmental health, animal health, public health

Emerging vectorborne and zoonotic diseases and public health consequences of environmental degradation have led to calls for One Health approaches that integrate public, animal, and environmental health perspectives and expertise (*1*,*2*). Recognizing the need to focus on One Health issues and priorities, the Southeastern Regional Center of Excellence for Emerging Infections and Biodefense (www.serceb.org) convened a conference January 30–31, 2012, to discuss development of a research and education framework for One Health.

For this conference, organizers defined One Health as the collaborative efforts of multiple disciplines working locally, nationally, and globally to attain optimal health for humans, domestic animals, wildlife, plants, and the environment (*3*,*4*). Objectives were to 1) identify issues relevant to developing a framework for a comprehensive research and education agenda for One Health and 2) strengthen relationships among participants from organizations in the southeastern United States. A planning committee developed the agenda and identified a diverse group of participants from academic, federal, state, and nongovernment organizations involved in One Health. Peter Daszak (EcoHealth Alliance) provided a global perspective on One Health, and Lisa Conti (One Health Solutions) provided a southeastern United States perspective in plenary presentations; these presentations were followed by work groups that focused on research and education themes. These themes were agribusiness and food safety; emerging disease surveillance and pathogen discovery; effect of ecologic and environmental stress on human and animal health; predictive modeling of diseases; workforce development, education, and training priorities; interdisciplinary preparedness, response, and biosecurity; occupational and avocational issues; and logistics and potential funding streams. Report sessions provided attendees with opportunities to comment on summaries of each group’s discussions.

The group identified 2 broad One Health research issues: 1) the need to develop and evaluate interventions with the potential to provide economic benefits to human or animal health to demonstrate a return on investment and 2) the need to engage behavioral science researchers and social marketers in conducting research to better understand consumer perspectives on One Health issues. The need to develop rapid diagnostics for zoonotic pathogens for use in the field and at the point of care was emphasized. Longitudinal environmental data acquisition is needed to support predictive modeling focused on the implications of changes at the human–animal–environment interfaces. Examples of identified research gaps in predictive modeling of diseases at these interfaces included data availability (cases, reservoirs, wildlife ranges), data granularity (land cover), real-time data to incorporate into models to support public health decision making, and lack of repositories for animal and human diagnostic specimens and pathogens. The need for interdisciplinary research teams to address these priorities was emphasized. The need to involve representatives of the food industry in identifying specific research priorities and the design of protocols was highlighted as a way to improve collaboration and communication and build trust.

Research issues identified as of particular relevance in the southeastern United States included antimicrobial drug use and resistance in poultry and livestock and occupational hazards in the agricultural and food animal sectors and among migrant workers. Additional examples of regional issues included the possible health effects of rising sea levels caused by climate change; food safety; raccoon rabies; vector-borne diseases (e.g., dengue, West Nile encephalitis, and eastern equine encephalitis); and the implications of the release of exotic pets on disease risk.

The One Health education agenda discussed by the group focused on implementing early exposure to One Health concepts in science classes in secondary schools, colleges, and universities; engaging professional societies; embedding training opportunities within industry; and using social media and networking tools. Although many disciplines could contribute to and benefit from One Health curricula and training programs, until recently no formal academic training program was dedicated to One Health training. Universities in the southeast are leading the way in education and training in One Health. The University of Florida has developed the first training program, a Masters in Health Science in One Health, and has introduced a doctoral degree program in Public Health with a concentration in One Health (*5*). The University of North Carolina’s Institute for Global Health and Infectious Diseases has a One Health lecture series to enhance collaborations between physicians, veterinarians, researchers, and other local and global health professionals (*6*). North Carolina State University’s College of Veterinary Medicine views One Health activities as a priority and provides opportunities for its students to interact with such groups as the North Carolina One Health Collaborative and Center for Comparative Medicine and Translational Research (*7*). Internationally, further expansion of efforts, such as Field Epidemiology and Laboratory Training Programs (*8*) and other training initiatives to include veterinary and environmental health trainees, was identified as a critical component of a One Health education agenda. Conference attendees concluded that 1) educational efforts also should focus on science teachers, funding agencies, and the public; 2) educational programs are needed for workers who have occupational exposures to livestock and wildlife about risk factors for infection and prevention strategies for specific infections; and 3) educators; legislators; and federal, state, and local officials should be informed of the added value of One Health approaches.

Several cross-cutting research and education priorities were emphasized throughout the work group discussions. These included the need to demonstrate the economic benefit of One Health approaches; engender collaboration and trust among a broad range of disciplines and sectors; emphasize professional and public education regarding One Health; focus on the changing human–animal–environment interface; and develop, assemble, and share data on human, animal, and environmental health issues. The One Health approach is by definition transdisciplinary; thus, many professionals would benefit from One Health curricula. One Health research could be furthered by funding support for transdisciplinary research projects and education. Documentation of the economic benefits of successful One Health collaborations is critical. Finally, the need to link One Health approaches with added value to national strategies, such as the National Prevention Strategy (*9*), Healthy People 2020 (*10*), the National Security Strategy (*11*), and the National Strategy for Biosurveillance (*12*), was emphasized.
